# Effect of irrigation on rural transformation and income at the district level in Bangladesh

**DOI:** 10.1371/journal.pone.0326504

**Published:** 2025-06-18

**Authors:** Subrata Saha, Mohammad Jahangir Alam, Al Amin Al Abbasi, Ismat Ara Begum, Maria Fay Rola-Rubzen, Panam Parikh, Andrew M. McKenzie

**Affiliations:** 1 Department of Agribusiness and Marketing, Bangladesh Agricultural University, Mymensingh, Bangladesh; 2 Department of Economics, Mawlana Bhashani Science and Technology University, Tangail, Bangladesh; 3 Department of Agricultural Economics, Bangladesh Agricultural University, Mymensingh, Bangladesh; 4 Center for Agricultural Economics and Development, UWA School of Agriculture and Environment, University of Western Australia, Australia; 5 Nutrition for Impact, Singapore, Singapore; 6 Department of Agricultural Economics and Agribusiness, University of Arkansas, Fayetteville, United States of America, University of Arkansas, Fayetteville, United States of America; World Health Organization, Regional Office for South-East Asia, INDIA

## Abstract

The study assesses the effect of investment in irrigation on rural transformation (RT) and rural income in Bangladesh. The analysis utilizes panel data from four rounds of nationally representative Household Income and Expenditure Surveys from 2000 to 2016. The share of irrigated land serves as an indicator of the level of irrigation investment in irrigation infrastructure. The study employs both ordinary least squares and fixed effects regression models. The findings indicate that the share of irrigated land has a positive effect on RT, particularly on the share of high-value agriculture and the share of rural non-farm employment. Additionally, the share of irrigated land positively affects rural income. Moreover, the study reveals that the effect of irrigation is positive at all stages of RT, especially for the share of high-value agricultural commodities. However, the impact of irrigation on the share of rural non-farm employment and rural income is inconclusive across different stages of RT. Overall, our results underscore the importance of prioritizing the construction of irrigation infrastructure by the government and encouraging the private sector to invest in irrigation.

## 1. Introduction

Rural areas in Bangladesh confront a myriad of challenges, including limited resources, low agricultural productivity, unemployment, and limited access to basic services and infrastructure [1]. Recognizing these challenges, IFAD (2016) recommends investment in irrigation as a fundamental strategy to foster rural transformation (RT) [2]. Irrigation not only enhances agricultural productivity but also stimulates non-farm employment opportunities and generates income in rural communities [3]. Irrigation boosts agricultural productivity, creating surplus production that encourages trade, and services. The increased agricultural output generates demand for labor in transportation, storage, and marketing, leading to the creation of non-farm employment and income generation through enhanced agricultural productivity, increased labor demand, and rising local consumption. Given Bangladesh`s dense population and heavy reliance on agriculture, irrigation plays a critical role in bolstering agricultural output to meet the dietary needs of its vast population. Despite being endowed with a deltaic landscape, abundant rivers, and significant rainfall, the country experiences erratic precipitation patterns, underscoring the necessity for continuous water sources to sustain agriculture.

RT in Bangladesh involves complex economic, social, and political changes within rural landscapes. This transformation is characterized by the shift from agrarian economies to a more diversified rural economy, incorporating both high-value agriculture and non-farm employment [2]. Agricultural policies, rural development strategies, responses to natural disasters, and the diversification of rural employment and the economy have all shaped this transformation [4–6]. However, irrigation is the most important development strategy in transforming the rural economy of Bangladesh and has played a crucial role in diversifying employment from the agricultural sector to non-agricultural sectors [2].

Since independence in 1971, the government of Bangladesh has undertaken significant efforts to enhance irrigation infrastructure and implement initiatives such as the Flood Action Plan (FAP) to address floods, droughts, and agricultural water needs [7–9]. Over time, Bangladesh’s irrigation systems have transitioned from solely government-controlled to incorporating public-private partnerships (PPPs) aimed at improving efficacy and efficiency [1]. While the government continues to provide policy guidance, technical support, and financial aid, there has been a shift towards greater involvement of producers and local communities in irrigation management [1,9]. Changes in government policies, such as lifting the ban on private sector investments, promoting imports of agricultural machinery, reducing import duties on irrigation accessories, and eliminating equipment standardization restrictions, have significantly expanded irrigation systems across Bangladesh. This liberalization has resulted in substantial investments in groundwater irrigation development, leading to enhanced productivity and positive impacts on rural livelihoods nationwide [1].

Understanding the intricate relationships between rural transformation and irrigation is essential. The expansion of irrigation enables farmers to shift from less labor-intensive grain production to more labor-intensive cash crops such as fruits, livestock, and aquaculture. This shift can significantly impact employment in both agricultural and non-farm sectors. This study contributes to the existing literature by providing a region-level empirical assessment of the impact of irrigation on rural transformation and income across districts in Bangladesh. Most existing studies either focus on national-level data or fail to fully integrate the multidimensional aspects of RT. Moreover, our study uniquely incorporates panel data across four time periods (2000–2016), allowing for a dynamic understanding of these relationships. By utilizing the Rural Transformation Index, this research categorizes districts into different stages of transformation, offering a nuanced perspective on how irrigation affects each stage. This differentiation provides targeted insights for policymakers aiming to optimize irrigation investments at the district level. Additionally, the study examines the differential impact of irrigation on high-value agriculture, non-farm employment, and rural income, presenting a comprehensive view of how irrigation contributes to sustainable rural development.

The study of irrigation in Bangladesh is crucial, as the country has significant water resources and relies heavily on agriculture. This study addresses existing gaps in the literature on RT and irrigation investment. It contributes to existing knowledge by providing a region-level empirical assessment of irrigation’s impact on various dimensions of RT and rural income in Bangladesh. First, it offers comparative assessments of irrigation investment across regions, addressing the lack of studies evaluating the effects of irrigation on RT and rural income within different regions of the country. Second, it extends the analysis beyond mere productivity enhancements to explore how irrigation investments influence the cultivation of high-value commodities, such as fruits, vegetables, aquaculture, and livestock. Third, it examines how irrigation affects non-farm employment patterns, shedding light on how changes in water availability drive rural economic diversification. Fourth, by analyzing per capita rural income from agricultural and non-agricultural sources, this study provides a more comprehensive picture of irrigation’s role in fostering broader socio-economic development. By capturing these distinct yet interconnected aspects of RT, the research not only fills a critical gap in the existing literature but also offers insights that can inform targeted policies and investments for sustainable RT. Moreover, this study contributes to the underexplored area of irrigation’s impact on rural transformation and income in the specific context of Bangladesh, providing valuable insights for policies and investments aimed at promoting sustainable rural development. In addressing these multiple dimensions, this research fills a critical gap in the literature and provides valuable implications for both regional and national policy frameworks.

## 2. Literature review

Evidence from various countries demonstrates the critical impact of irrigation management on RT and food security and with economic and social advancements in rural areas, there is a need to expand the role of irrigation in driving more sustainable and inclusive growth [10].

High-value crops often require more controlled and precise irrigation along with a higher watering frequency, which can enhance both crop quality and water efficiency [11]. For example, in the Philippines, there is evidence of farmers benefiting from canal irrigation by expanding non-farm activities and livestock production [12]. Irrigation enhances agricultural productivity, reducing the time and labor required for farming and enabling farmers to diversify their sources of income. As a result, non-farm activities expand. Moreover, the increased water availability from irrigation systems can facilitate the development of other revenue-generating enterprises, including small-scale agro-processing and related trade and service-based activities. This diversification is crucial for rural livelihoods as it acts as a safeguard against the inherent risks of agricultural production, such as crop failure and fluctuating market prices. Similarly, investment in irrigation in Nepal has resulted in significant boosts in agricultural output and rural household income, particularly for smallholder farmers [13]. In summary, it is evident that irrigation is closely linked to two types of RTs: modifications in the agricultural sector and shifts from farm to non-farm employment [2,3,14].

Oluniyi and Bala (2021) reported that participatory irrigation management and other government initiatives proved effective in improving food security and advancing agricultural development in Nigeria [15]. Their findings emphasized the need to redirect from private to public irrigation development and management in Nigeria. Similarly, Ahmed (2019) examined the positive effects of irrigation on food security in rural farming households in Ethiopia, noting that it helped alleviate poverty in rural areas [16]. Rahman & Mondal (2015) highlighted the significance of enhancing irrigation efficiency and water productivity through a combination of surface and groundwater sources [17]. They also stressed the importance of preserving agricultural land through the Land Use Act and promoting research and innovative ideas to improve crop yields, all of which contribute to maintaining food security. In another study, Rahman & Parin (2009) highlighted the beneficial impact of expanding irrigated land, including groundwater and surface water sources, and adopting modern technologies on food grain production in Bangladesh [18]. Furthermore, the implementation of supportive policies and assistance for farmers was identified as a crucial element in achieving self-sufficiency in food grains. The lack of access to irrigation water in rural communities has been reported to hinder their ability to combat food insecurity, reduce rural youth unemployment, and increase the participation of women in various agricultural activities [19]. Therefore, we propose our first two hypotheses as follows:

**H1:** The impact of irrigation is positively associated with rural transformation at the regional level in Bangladesh.

**H2:** The effect of irrigation on rural transformation is positive at different stages at the regional level in Bangladesh.

The limited literature investigating the effects of user-managed small-scale irrigation also highlights its positive effect on rural income, poverty alleviation, and the transformation of rural areas [12,20,21]. Evidence from Bangladesh demonstrated that the development of irrigation not only raised the incomes of households and the wages of workers but also improved the overall quality of life in rural areas and boosted non-farm activities [22]. However, RT is also influenced by the wage rate of rural labor in non-farm employment, the price ratio of high-value and staple agricultural commodities, and the price index of agricultural inputs [23]. The wage rate of rural labor in non-farm employment can reflect the opportunity cost of labor for farmers. If the wage rate of non-farm employment is high, farmers may prefer to work in the non-farm sector rather than on their farms [24]. Similarly, in regions where high-value goods fetch higher prices compared to other crops, farmers may be incentivized to grow more high-value goods. Regardless of the amount of irrigated land available, this could lead to a greater share of high-value agricultural commodities [23]. The price index of agricultural inputs represents the cost of production for farmers and includes expenses such as seeds, fertilizers, pesticides, and labor. If the price of inputs rises, farmers may be less inclined to invest in high-value commodities or may switch to crops that require fewer inputs [24]. In such instances, it is expected that irrigation may have a limited effect on RT and warrants further investigation. Based on this information, we formulate the third and fourth hypotheses as follows:

**H3:** The price ratio, non-farm wage, and the price index of agricultural inputs are positively associated with rural transformation at the regional level in Bangladesh.

**H4:** Irrigation has a positive impact on per capita rural income at the regional level in Bangladesh.

Existing studies have explored the relationship between irrigation and key development issues such as poverty alleviation, food security, income inequality, and rural households [25–27]. However, some focus on the effects of rural transformation on gender, poverty, and inequality, yet they do not specifically address the role of irrigation in these transformations [28–30]. Additionally, few studies consider aspects of rural transformation such as urbanization, non-agricultural income, non-farm employment, and rural infrastructure [31–33]. A comprehensive analysis of the impact of irrigation on rural transformation across regions in a specific country is still lacking. Thus, there is a significant gap in research connecting irrigation investments with broader rural transformation processes, both globally and nationally, particularly in Bangladesh. Furthermore, there is a lack of panel data studies at the district level to explore this relationship. Such a district-level approach could provide valuable insights into the regional disparities and dynamics of these variables over time, helping to inform more targeted policy interventions. This gap is particularly evident in developing countries like Bangladesh, where the interplay of economic, social, and cultural factors across regions complicates the analysis.

### 2.1. Theoretical perspectives of the study

This study is grounded in several theoretical frameworks to examine the impact of irrigation investment on rural transformation and rural income in Bangladesh. Rural development, agricultural reforms, and diversification are often considered preconditions for industrial development [34], with industrial and agrarian revolutions historically progressing in tandem [35]. Economic growth in rural areas is not solely driven by agricultural transformation but also by the expansion of non-agricultural sectors a process facilitated by irrigation [2,36].

Irrigation plays a critical role in driving agricultural output and enabling economic diversification. Recognized as a key driver of rural development, it directly enhances livelihoods and incomes by improving crop yields and agricultural productivity [37]. It also stabilizes production, particularly in drought-prone areas, mitigating climate variability and environmental challenges [38]. Additionally, irrigation systems contribute to higher incomes and improved food security [37]. By ensuring reliable water access, irrigation increases crop yields, allowing rural households to sell surplus crops in local or regional markets, thereby boosting income [39]. Enhanced agricultural productivity further stimulates the rural economy by generating employment in sectors such as processing, storage, and transportation [40].

Irrigation promotes agricultural intensification and diversification by enabling multiple cropping cycles, increasing yields, and fostering a transition to high-value crops such as fruits and vegetables. It also improves market access [41–43]. This agricultural expansion strengthens forward linkages with agro-processing and backward linkages with input suppliers, fostering rural non-farm employment [44]. Moreover, the multiplier effects of rising agricultural income drive demand for non-farm goods and services, further stimulating economic growth [45].

## 3. Methodology

### 3.1. Data sources

We use data from the Household Income and Expenditure Survey (HIES) conducted by the Bangladesh Bureau of Statistics (BBS) in 2000, 2005, 2010, and 2016. The analysis comprises 128 district-level observations on RT, irrigation, and three control variables (wage rate of rural non-farm labor, the price ratio between high-valued and all (total) agricultural commodities as a group, and the price index of agricultural inputs) from 32 districts in Bangladesh. Only districts with available data on the share of irrigated land were included in the analysis. Investment in irrigation was gauged using the share of irrigated land obtained from the Yearbook of Agricultural Statistics (Bangladesh Bureau of Statistics, 2000–2016). To assess rural transformation, our study follows the approach of Huang & Shi (2020) and Rola-Rubzen et al. (2024), using two indicators: RT1, which quantifies the share of high-value agricultural production (e.g., vegetables, fruits, fish, and livestock), and RT2, which measures the share of non-farm employment in rural areas [28,36]. To measure irrigation investment, we use the share of irrigated land, consistent with research by Shi (2022) and Shi & Huang (2023) conducted in China [23,46].

A correlation analysis was performed to ascertain the relationship between the share of irrigated land (an independent variable) and RT1 and RT2 (dependent variables). Furthermore, this study illustrates the stages of RT by examining the correlation between two RT indicators, RT1 and RT2, as outlined in the next section. Table 1 presents an overview of the variables used, along with their corresponding definitions and units of measurement.

**Table 1:** Definitions of variables and sources.

Variables	Definition	Sources	Units
The share of high-value agricultural commodities	The proportion of total output values of vegetables, fruits, fish, livestock, and spices in gross agricultural output values	HIES data (BBS)	Proportion
The share of rural non-farm employment	The proportion of rural laborers engaged in non-farm activities	HIES data (BBS)	Proportion
Per capita rural income	Per capita gross income of rural households. It is measured in real terms at a constant price of 2010.	HIES data (BBS)	Bangladeshi Taka (BDT)
The share of irrigated land	The ratio of the irrigated areas to the total cultivated areas	Yearbook of Agricultural Statistics (BBS)	Proportion
The wage rate of rural non-farm labor	The wages of individuals who are engaged in non-agricultural economic activity in rural areas	HIES data (BBS)	Bangladeshi Taka (BDT)
The price ratio between high-value agricultural commodities to total agricultural commodities	The price ratio between high-value agricultural commodities and all agricultural commodities as a group referred to as total agricultural commodities	HIES data (BBS)	Proportion
The price index of agricultural inputs	By dividing the weighted average of each year’s agricultural input prices by the weighted average of the base year’s agricultural input prices	HIES data (BBS)	Proportion

The wage rate used in this analysis is the weighted average wage for rural non-farm laborers, derived from the HIES. It aggregates wage data from various non-agricultural sectors, including agriculture-related services, retail, and business labor, capturing the diversity of rural employment. The price ratio of high-value agricultural commodities to total agricultural commodities is calculated by dividing the weighted average price of high-value products (e.g., fruits, vegetables, fish, and livestock) by the weighted average price of all agricultural commodities. The price index is a weighted average of agricultural input prices, with weights reflecting each input’s share in total agricultural expenditure at the district level. It measures price levels relative to the base year (2010).

### 3.2. Stages of RT

In this research, we employ Principal Component Analysis (PCA) to construct a Rural Transformation Index (RT index) by incorporating two RT indicators, RT1 and RT2. PCA is a statistical technique for multivariate analysis, commonly used in exploratory data analysis and predictive models. This approach reduces the dataset’s dimensionality while retaining the maximum variance, thus enabling a more comprehensive understanding of the primary trends associated with rural transformation. By integrating RT1 and RT2, the RT index effectively encapsulates the intricate and multifaceted nature of rural transformation across districts in an efficient manner. Appendix A presents the development of the RT Index in Bangladeshi districts from 2000 to 2016.

Using the RT index, we categorized the 128 observations gathered from 32 districts in Bangladesh into three distinct stages of rural transformation—low, medium, and high. Let dX represent the difference between the minimum (Xmin) and maximum (Xmax) values across all districts in terms of the RT index. All districts are categorized as follows based on dX/3: low RT index (RT index <(Xmin + dX/3), medium RT index (Xmin + dX/3 < RT index < Xmin + 2dX/3), and high RT index (RT index> (Xmin + 2dX/3) [36,47]. This classification provides a comprehensive insight into the progression of rural transformation across different regions. A low RT index indicates that high-value agriculture and non-agricultural employment in rural areas is relatively underdeveloped in a given district. To spur economic development, these districts require targeted interventions to enhance agricultural productivity and expand non-agricultural employment opportunities. Conversely, districts with a medium RT index demonstrate moderate progress, suggesting they are in a transitional phase. Despite the effective implementation of certain strategies, these areas require further support to fully realize their potential. In conclusion, districts characterized by a high RT index exhibit significant progress in rural non-agricultural employment and high-value agriculture, indicating substantial rural transformation.

This categorization not only assists policymakers in identifying districts at the forefront of rural transformation while others are lagging but also facilitates the customization of development strategies tailored to each category. For instance, districts with a low RT index could benefit from implementing comprehensive agricultural reforms, enhancing market accessibility, and developing infrastructure to foster non-agricultural employment prospects. Meanwhile, districts with a medium RT index would require policies that amplify and sustain support for ongoing initiatives, ensuring the acceleration and sustainability of progress. Conversely, districts with a high RT index may seek to maintain their growth trajectory by promoting higher-value agricultural and industrial activities, fostering innovation, and improving value chains. This methodical and evidence-based approach makes the overarching goal of achieving sustainable and equitable rural transformation throughout Bangladesh more attainable.

The RT index serves as a metric for tracking the evolution of agricultural sectors, reflecting the shift from predominantly low-value agricultural production to higher-value outputs, as well as the transition from employment largely dependent on traditional agriculture to a more diversified non-agricultural workforce within districts. In 2000, most districts had negative RT scores, classifying them under Stage 1, indicating minimal diversification and resilience. Over time, a clear trend of progress has emerged, with many districts advancing to Stage 2 and ultimately reaching Stage 3 by 2016, as shown in Appendix A. This progress reflects a significant increase in high-value agricultural production and non-farm employment in rural areas, signaling greater economic diversification and resilience at the district level. The movement from one stage to the next shows the transformation of rural Bangladesh from being largely based on traditional agriculture to having a wider range of non-farm activities and enhanced resilience.

### 3.3. Econometric model specification

The study applies ordinary least squares and fixed effects panel regression to analyze the relationship between RT and irrigation and control variables [48]. The selection of these models is grounded in existing literature, with ordinary least squares being a commonly used method for modeling RT [28,49]. Additionally, a fixed effects model is used to control for time-invariant unobserved heterogeneity at the district level, ensuring that the estimated effect of irrigation is not biased by unobserved, district-specific factors [30,50,51]. To capture the impact of irrigation on three different stages of RT, the study incorporates an interaction term for irrigated land and a group of stage dummies. Furthermore, a fixed-effect regression model is adopted to address time-invariant, unobserved individual traits potentially associated with the observed independent variables [52]. The fixed-effect model controls for unobserved time-invariant factors, while the fixed-effect model with interaction terms elucidates how the share of irrigated land influences RT1 and RT2 at different stages of RT. In our econometric approach, we applied a fixed-effects model that incorporates both time-fixed effects and district-fixed effects. This enhancement allows us to isolate and examine the impact of irrigation while accounting for both time and district-specific variations.

Impact of irrigation on RT


$${RT}_{ij}=\;\alpha_{i\;}+\;\alpha_{2\;}{IR}_{ij}+\;\alpha_{3\;}W_{ij}+\;\alpha_{4\;}{Ph\_ptotal}_{ij}+\alpha_{4\;}{Pindex}_{ij}+u_{i}$$ (1)

$${RT}_{ij}=\alpha_{i\;}+\;\alpha_{2\;}{IR}_{ij}+\alpha_{3\;}W_{ij}+\alpha_{4\;}{Ph\_ptotal}_{ij}+\alpha_{4\;}{Pindex}_{ij}+u_{i}+\tau_{j}$$ (2)

$${RT}_{ij}=\alpha_{i\;}+\;{\alpha_{2\;}IR}_{ij}*\;D_{ijt}+\alpha_{3\;}W_{ij}+\alpha_{4\;}{{Ph}_{ptotal}}_{ij}+\alpha_{4\;}{Pindex}_{ij}+u_{i}+\tau_{j}$$ (3)

Where RT is expressed as a vector of the share of high-value agricultural commodities in agricultural output value (Model 1), the share of non-farm employment in rural labor employment (Model 2), and rural income per capita (Model 3). Equation 1, i represents the district, and j represents the year. “IR” represents the share of irrigated land; and “W”, “Ph_ptotal”, and “Pindex” indicate the wage rate of rural non-farm labor, the price ratio between high and total agricultural commodities, and the price index of agricultural inputs, respectively. Equation 2 employs a fixed effect model, while Equation 3 uses a group of stage dummies to analyze the effects of irrigation on RT at different stages.

Here, $D_{IJt}$ represents a set of dummies indicating whether district i is in the lower (t = 1), middle (t = 2), or higher (t = 3) stage of year j. If the coefficient $\alpha_{2\;}$ is statistically significant, it will support the sequential hypothesis, suggesting that the impact of irrigation at a higher stage of RT is greater than at a lower stage of RT. In other words, the study is testing whether the impact of irrigation is greater in the later stages of RT compared to the early stages of RT. By identifying which stages of RT are most influenced by irrigation, policymakers can focus their efforts and allocate resources accordingly, aiding rural areas in growing their economies in a manner that benefits everyone in rural areas.

This study uses moments-quantile regression (MM-QR) with fixed effects, following the technique outlined in the study by [53]. We compared these results with those obtained using the FEM. The MM-QR methodology provides a comprehensive analysis of the impact of independent variables on the entire conditional distribution of the dependent variable, in contrast to conventional methods. The MM-QR model with panel fixed effects is developed following the conditions outlined by [54].


$${QC}_{ij}\left(\eta |D_{ij}\right)=\;{\text{ʓ}}_{i}(\eta )+{\overset{´}{D}}_{ij}\beta +\Psi_{ij}\gamma q(\eta with\;{\text{ʓ}}_{i}(\eta )\equiv \left({\text{ʓ}}_{i}+\theta_{i}q(\eta )\right)$$ (4)

Here, C and D are the dependent (RT1 and RT2) and IR, W, Ph_ptotal, and Pindex are the independent variables. $\Psi_{ij}$ is a vector of known differentiable transformations of the components of D. ${\text{ʓ}}_{i}(\eta$ is the quantile (η) fixed effect for panel unit i at time j, while β and γ are the parameters to be estimated in Equation 4.

To address the potential endogeneity of the irrigation variable, the study employs a fixed effects regression model. This approach helps mitigate concerns about omitted variable bias and reverse causality [55,56]. In the context of irrigation and rural transformation, reverse causality may arise, as RT itself can influence the extent of irrigation investment. To account for this, we use fixed effects regression to control for unobserved time-invariant heterogeneity at the district level, which could confound the relationship between irrigation and rural transformation. The fixed effects model enables us to isolate the causal effect of irrigation by controlling for district-specific, time-invariant factors, such as districts that may influence RT but are not directly captured by observable variables. This approach is crucial, as OLS estimates might reflect only a correlation between irrigation and rural transformation, rather than a causal relationship. By using fixed effects, we focus on how changes within districts over time (rather than across districts) affect the outcomes, ensuring that the observed relationships are not driven by unobserved, time-invariant factors.

The fixed effects model is applied to analyze the impact of the share of irrigated land on the two rural transformation indicators (RT1 and RT2), as well as on rural income. We incorporate interaction terms between irrigation and stage dummy variables into the model to examine the effect of irrigation at different stages of RT.

We focus exclusively on the FEM due to its ability to account for time-invariant, district-specific variables that may induce endogeneity in our analysis. This approach enables a more precise assessment of the effects of irrigated land by controlling potential biases that remain constant over time within the same district. By focusing on the FE model, we ensure that our analytical method is rigorously aligned with the study’s objectives. Furthermore, the choice of FEM is consistent with previous studies on rural transformations, reinforcing the robustness of our approach [53]. Additionally, we conducted regression-based endogeneity tests to check the endogeneity of our control variables- namely, the wage rate of non-farm work in rural areas, the price ratio between high-value and all agricultural commodities, and the price index of agricultural inputs. The tests revealed that the residuals from these regressions are not statistically significant, indicating that endogeneity is not an issue in our dataset.

## 4. Results and discussion

### 4.1. Descriptive statistics

Table 2 presents summary statistics for the RT, irrigation, and control variables across the four time periods. The share of high-value agricultural commodities in Bangladesh increased from 9.32% in 2000 to 16.96% in 2016. A similar upward trend was observed in rural non-farm employment, with the share rising from 37% to 57% between 2000 and 2016. This trend aligns with reports from Indonesia, which also noted increases in both the shares of high-value agricultural commodities and rural non-farm employment between 1999 and 2019 [57]. The consistent growth in both RT indicators from 2000 to 2016 suggests that Bangladesh, akin to Indonesia, is undergoing a significant transition towards becoming predominantly non-agricultural. The share of irrigated land also surged by approximately 79.63% during the same period, from 27.84% to 50%, indicating increased investment in irrigation over time.

**Table 2:** Summary statistics of RT, irrigated land and control variables.

Variables	Total (N = 128)		2000 (N = 32)		2005 (N = 32)		2010 (N = 32)		2016 (N = 32)	
	Mean	St. Dev.	Mean	St. Dev.	Mean	St. Dev.	Mean	St. Dev.	Mean	St. Dev.
Share of high-value agricultural commodities (%)	12.62	3.83	9.32	1.87	10.84	2.14	13.34	2.78	16.96	3.14
Share of rural non-farm employment (%)	46.51	8.18	37.09	3.41	42.56	3.34	49.59	3.63	56.79	3.36
Per capita rural income	8788.41	3568.3	5268.21	1104.5	7230.06	1659.17	9678.5	2641.9	12976.8	2643.2
Share of irrigated land (%)	37.79	9.51	27.84	4.43	32.97	4.64	40.36	4.94	50.01	4.21
Price ratio of high valued to total agricultural commodities (%)	61.81	8.18	52.76	3.05	57.41	3.07	64.39	3.03	72.69	3.54
Wage rate of rural labor non-farm employment	200.15	92.55	101.86	6,69	146.98	16.15	213.1	26.74	338.64	34.71

Furthermore, the wage rate of rural labor in non-farm employment, averaging TK 101 in 2000, nearly tripled by 2016. Additionally, the price ratio of high-valued to total agricultural commodities exhibited a 38% increase between 2000 and 2016.

### 4.2. Trends in the shares of high-value agricultural commodities and rural non-farm employment

**Fig 1:**
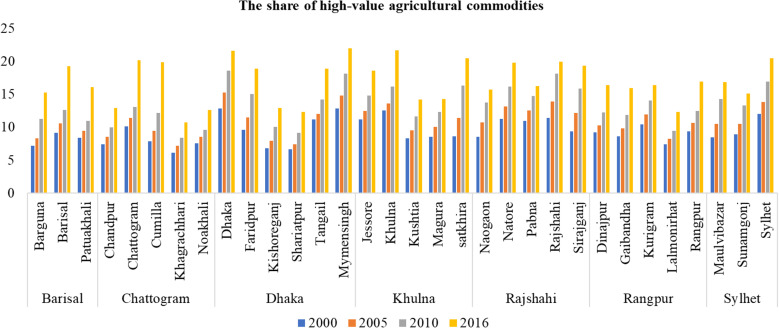
The share of high-value agricultural commodities across all districts. **Note(s):** The raw data used to estimate per capita rural income are from the Yearbook of Agricultural Statistics and Household Income and Expenditure Survey (HIES) (various years) published by the Bangladesh Bureau of Statistics (BBS).

Fig 1 presents the share of high-value agricultural commodities in Bangladesh for the years 2000, 2005, 2010, and 2016, grouped by the country`s seven divisions: Dhaka, Chattogram, Barisal, Khulna, Rajshahi, Rangpur, and Sylhet. The analysis reveals several noteworthy observations regarding RT1 (Fig 1). Firstly, an analysis of agricultural sector transitions in Bangladesh at the regional level revealed a notable shift from low-value to high-value crops. However, this transition was not uniform across all districts. Variations in the adoption rates and initial levels of high-value agricultural commodities highlight the uneven progression of agricultural growth. These disparities may stem from differences in regional infrastructure, market access, agricultural regulations, and local economic conditions. Understanding these factors is crucial for developing tailored strategies that address the unique needs and capacities of each region, ensuring more equitable growth in the agricultural sector. The disparity arose as some districts adopted and produced high-value agricultural products—such as vegetables, fruits, fish, and livestock—more rapidly than others. The varying growth rates among districts can be attributed to differences in access to new technologies, timely financial resources, and the effectiveness of development plan implementation, which results in some districts making faster progress than others. This aligns with the findings of Hossain (2010) and Sarker (2016) [58,59]. Secondly, we found that in general, the transformation was much faster in the later period compared to the earlier period. Improvements in technology, infrastructure, market access, knowledge sharing, and increased efficiency in production and distribution among districts may have accelerated the transition from low-value to high-value agricultural commodities [60]. Thirdly, despite a large variation in the share of high-value agricultural commodities in 2000, we observed many districts with lower levels of high-value agricultural commodities catching up over time. The convergence of high-value agricultural commodities in districts with lower levels may be attributed to targeted government policies, increased credit availability, improved irrigation facilities, and extension services, as well as the implementation of targeted government interventions to promote high-value agriculture in underperforming districts. These findings are consistent with studies in China [61], India [62], and South Asia [63]. A similar trend has also been observed in China, where there has been a rise in the proportion of high-value agricultural commodities characterized by variations in production across China’s provinces [36].

**Fig 2:**
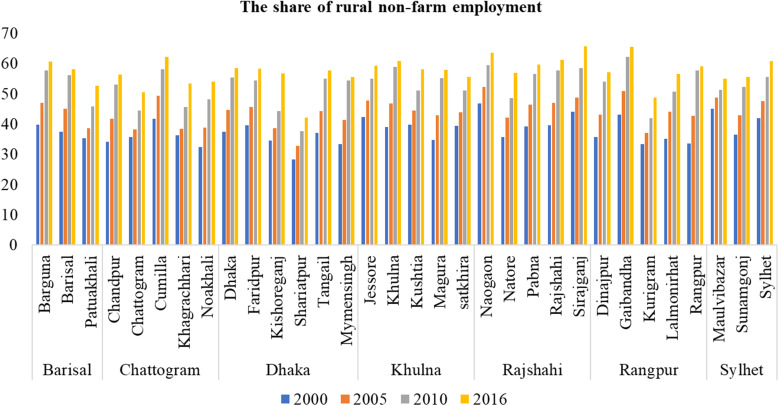
The share of rural non-farm employment across all district. **Note(s):** The raw data used to estimate per capita rural income are from the Yearbook of Agricultural Statistics and Household Income and Expenditure Survey (HIES) (various years) published by the Bangladesh Bureau of Statistics (BBS).

Fig 2 presents the trends of rural labor non-farm employment shares in 2000, 2005, 2010, and 2016. During the period 2000–2016, consistent growth was observed in the non-agricultural sector in all districts of Bangladesh, indicating a shift in the employment structure from that at the start of the twenty-first century [64]. This shift can be attributed to various factors, including the diversification of economic activities, education, growth of the service sector, asset ownership, access to credit, and supportive government policies [65,66]. The growth of non-agricultural sectors has been reported to help create new employment opportunities [67]. Overall, it was noted that the RT2 was faster in 2016 compared to preceding years and varied substantially across the divisions and districts. Dhaka and Chittagong divisions, which are the leading regions of the country, saw a significant rise in non-farm orientation. In contrast, the Rajshahi and Rangpur divisions showed the least growth in rural non-farm employment. This is consistent with Sen’s (2019) finding that the non-farm employment growth rate in the Rajshahi and Rangpur divisions is lower than the national average [68]. A similar increase in the share of rural non-farm employment has also been observed in Indonesia [57].

### 4.3. Trends in per capita rural income

**Fig 3:**
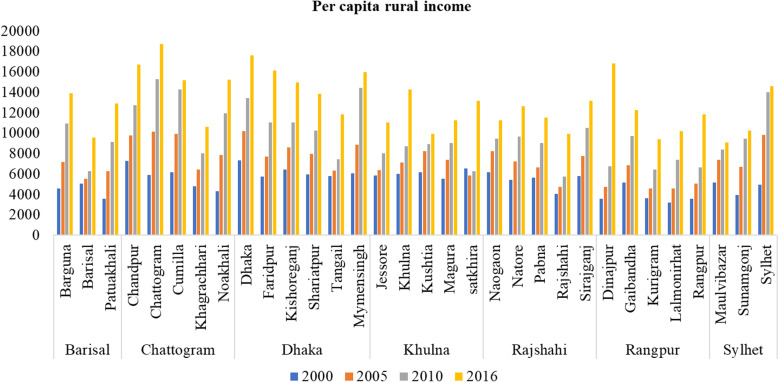
Per capita rural income by districts in 2000, 2005,2010 and 2016. **Note(s):** The raw data used to estimate per capita rural income are from the Household Income and Expenditure Survey (HIES) (various years) published by the Bangladesh Bureau of Statistics (BBS).

Fig 3 illustrates the increase in rural per capita income across all districts between 2000 and 2016. However, there existed a notable disparity in the growth rates among the districts. Specifically, Dhaka, Chattogram, Faridpur, Kishoreganj, Khulna, Rajshahi, Dinajpur, and Rangpur exhibited significantly higher income growth compared to Khagrachari, Mymensingh, Gaibandha, and Maulvibazar. This variation in growth rates may be attributed to regional heterogeneity in development plans, budget allocation and implementation, infrastructure and connectivity, local governance and administrative capacity, resource endowment, and inclusive development policies, as suggested by Mithun (2021) [69].

### 4.4. The trend in the share of irrigated land

**Fig 4:**
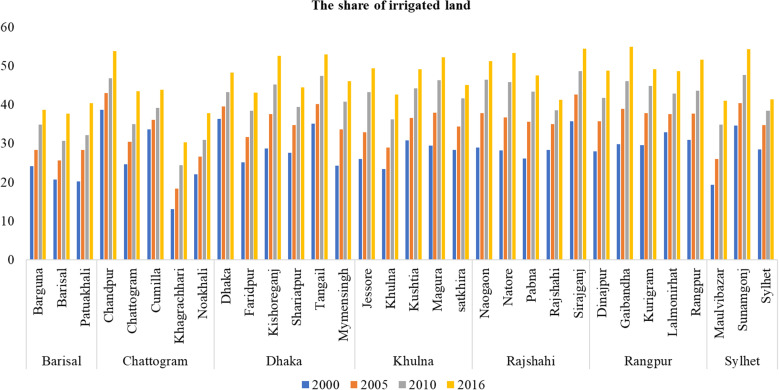
The share of irrigated land in all districts in 2000, 2005,2010 and 2016. **Note(s):** The raw data used to estimate the share of irrigated land are from the Yearbook of Agricultural Statistics (various years) published by the Bangladesh Bureau of Statistics (BBS).

Fig 4 illustrates an increasing trend in the share of irrigated land across all districts from 2000 to 2016. While diverse development trends were observed among districts, the overall pattern indicated a positive trend in the adoption and expansion of irrigation facilities. Certain districts in the Dhaka and Chattogram divisions exhibit higher growth rates in the share of irrigated land compared to others, highlighting regional disparities in the implementation and utilization of irrigation systems. Additionally, a transition from rainfed to irrigated systems was observed, indicating a notable improvement in agricultural practices. This shift holds the potential to mitigate agricultural drought occurrences in the country and facilitate initiatives aimed at promoting agricultural sustainability. Such initiatives may include the relocation of agricultural activities from vulnerable regions to more suitable places and the establishment of measures to safeguard cropland from the encroachment of urbanization [70]. This trend aligns with findings from a study conducted by Bai et al. (2022) in China [71].

### 4.5. Correlation between irrigation and RT

**Fig 5:**
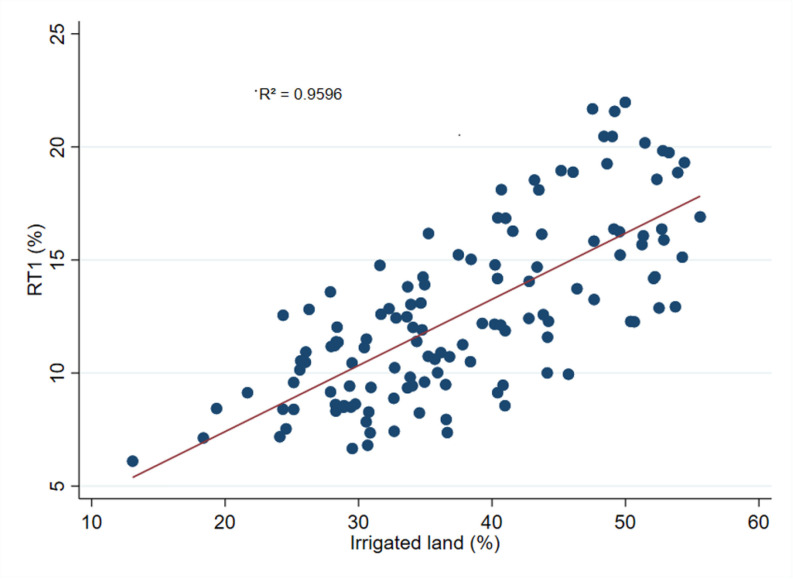
Correlation between the share of irrigated land and RT1. **Note(s):** The correlation between the share of irrigated land (%) and the share of high-value agricultural commodities (%) data in 32 districts between 2000 and 2016 is shown in Fig 5 The data on the share of irrigated land, and the share of high-value agricultural commodities were collected from HIES and the Yearbook of Agricultural Statistics of Bangladesh (various years) published by the Bangladesh Bureau of Statistics.

**Fig 6:**
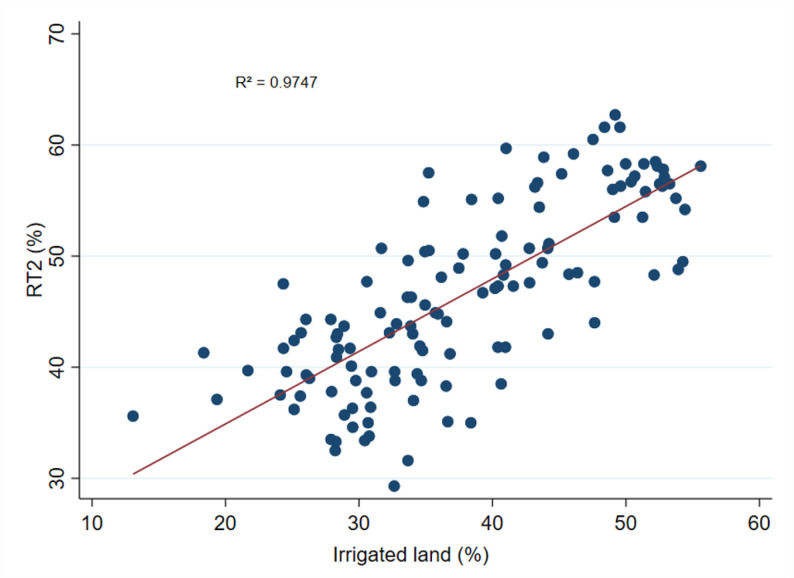
Correlation between the share of irrigated land and RT2. **Note(s):** The correlation between the share of irrigated land (%) and the share of non-farm employment in rural areas (%) in Bangladesh is depicted in Fig 6. The data on the share of irrigated land and the share of rural non-farm employment were collected from HIES and the Yearbook of Agricultural Statistics of Bangladesh (various years) published by the Bangladesh Bureau of Statistics.

**Fig 7:**
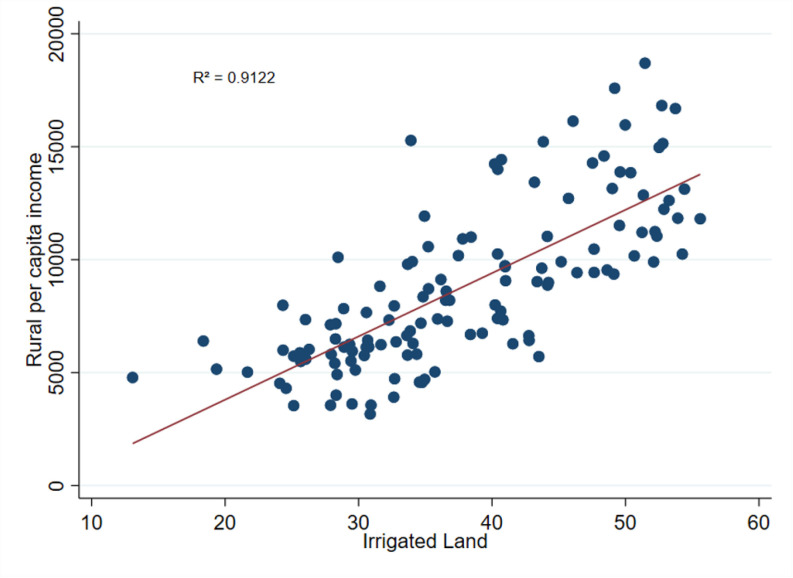
Correlation between the share of irrigated land and per capita rural income. **Note (s)**: From 2000 to 2016, this graph depicts the positive link between the share of irrigated land (%) and per capita rural income in 32 districts of Bangladesh. The data used to estimate the share of irrigated land and rural income came from the HIES and the Yearbook of Agricultural Statistics of Bangladesh (various years) published by the Bangladesh Bureau of Statistics.

Figs 5–7 show correlations based upon a simple OLS regression of RT1, RT2, and per capita income on the share of irrigated land across 32 districts in Bangladesh. In all cases, our results reveal a strong positive correlation. Fig 5 depicts a positive correlation between the share of irrigated land and the proportion of high-value agricultural commodities. Investments in irrigation infrastructure are known for the expansion of irrigated land, which is crucial for producing high-value agricultural commodities such as fruits, vegetables, fish, livestock, and spices [72]. These commodities are less reliant on rainfall and can be cultivated year-round, thus boosting agricultural output [73]. This finding is further supported by Reddy et al. (2017), who demonstrated that drip irrigation and fertigation techniques enhanced the growth and yield of various high-value commodities, including fruits and vegetables [74]. The R² value for Fig 5 is 0.9596, indicating a strong correlation between the share of irrigated land and the share of high-value agricultural commodities.

Similarly, Fig 6 illustrates a positive correlation between irrigation and rural non-farm employment, while Fig 7 indicates a positive correlation between irrigation and rural income. Irrigation can have spillover effects on the non-farm sector by contributing to increased productivity, which can lead to surplus labor in the agricultural sector and, consequently, greater employment opportunities in the non-farm sector [75]. Moreover, an increase in crop production resulting from irrigation can lead to an increase in the supply of raw materials for processing and manufacturing, thereby stimulating non-farm economic activity [76]. This scenario is consistent with the experience of the Philippines, where farmers benefited from canal irrigation and expanded into non-farm activities [12,22]. Additionally, other studies have shown that irrigation increases agricultural productivity [77], leading to an increase in farmers’ surplus income [78,79], which, in turn, contributes to the expansion of rural non-agricultural employment opportunities [80].

The R² values for 5, 6 and 7 are 0.9596, 0.9747, and 0.9122, respectively, indicating a strong correlation between the share of irrigated land and both the share of rural non-farm employment and rural per capita income. These high R² values suggest a robust and consistent relationship between the share of irrigated land, the RT indicators, and rural income.

### 4.6. Effect of irrigated land on RT1, RT2, and rural income

We conducted a regression analysis to examine the impact of the share of irrigated land on RT1 and RT2. Three models and their outcomes are presented in Table 3. These are the ordinary least squares (OLS) model, the fixed effect model (FEM), and the FEM with interaction terms for irrigation and stage dummies. Breusch and Pagan and Hausman’s tests indicated that the FEMs are better specified than the OLS. However, for completeness and to illustrate that our results are robust with respect to model specification we present the results for all three models. High R-square values for all 3 models indicate that the share of irrigated land, the wage rate of rural non-farm labor, the price ratio of high value to total agricultural commodities, and the price index of agricultural inputs account for a significant amount of the variation observed in RT1 and RT2.

**Table 3:** The impact of irrigation at different stages of RT.

	OLS	FEM		OLS	FEM	
	(1)	(2)	(3)	(4)	(5)	(6)
	RT1	RT1	RT1	RT2	RT2	RT2
IR_land	0.112^**^	0.193^***^	0.139^**^	−0.157	0.372^***^	0.358^***^
	(0.054)	(0.068)	(0.065)	(0.076)	(0.107)	(0.118)
ph_ptotal	0.022	0.094^*^	0.074^*^	0.236^**^	0.115	0.110
	(0.067)	(0.050)	(0.044)	(0.094)	(0.078)	(0.079)
Pinp_index	0.029	0.055^*^	0.028	0.167^**^	−0.032	−0.037
	(0.053)	(0.033)	(0.029)	(0.075)	(0.051)	(0.053)
NonFwage	0.030^*^	−0.003	−0.009	0.105^***^	0.086^***^	0.084^***^
	(0.017)	(0.009)	(0.008)	(0.024)	(0.014)	(0.014)
IR*D1			0.013^*^			0.003
			(0.007)			(0.013)
IR*D2			0.041^***^			0.009
			(0.008)			(0.014)
Year effect of IR						
2000		0.190^**^	0.151^**^		0.252^**^	0.271^**^
		(0.014)	(0.034)		(0.029)	(0.026)
2005		0.190^***^	0.145^**^		0.295^***^	0.314^***^
		(0.009)	(0.032)		(0.007)	(0.007)
2010		0.195^***^	0.140^**^		0.338^***^	0.359^***^
		(0.005)	(0.036)		(0.001)	(0.002)
2016		0.206^***^	0.141^**^		0.338^***^	0.357^***^
		(0.004)	(0.037)		(0.001)	(0.002)
_cons	−0.116^**^	−0.043	0.005	−0.327^***^	−0.161^***^	−0.150^***^
	(0.044)	(0.029)	(0.027)	(0.062)	(0.045)	(0.049)
*N*	128	128	128	128	128	128
*R* ^2^	0.578	0.941	0.956	0.817	0.977	0.977

*Notes: p-values in parentheses;* ^*^ *p < 0.1,* ^**^ *p < 0.05,* ^***^ *p < 0.01*

In the FEM model, the share of irrigated land was positively associated with RT1 (0.193, p-value < 0.003) and RT2 (0.372, p-value < 0.001). In contrast, in the OLS, the share of irrigated land was positively linked with RT1 but not with RT2. The positive impact of the share of irrigated land on RT1 suggests that irrigation can increase agricultural productivity and contribute to the production of high-value agricultural commodities. This may be because irrigation enables producers to grow crops year-round, thereby increasing crop yield and quality [81]. A positive correlation between irrigation and agricultural production has been previously reported in the literature [74,82]. However, the relationship between the share of irrigated land and RT2 may be more complex. Various non-agricultural factors, such as infrastructure development, market access, credit availability, and education levels, may differ across stages of RT and impact non-farm employment [51,83,84]. The positive influence of irrigation on RT2 seen in the FEM model is consistent with research that used rainfall and irrigation as proxy variables for agricultural productivity and found a strong link between an increase in agricultural productivity and the growth of informal (small-scale) manufacturing jobs [77]. By implementing a better irrigation system, agricultural growth can be boosted, and the negative effects of unpredictable rainfall can be lessened, making this sector less vulnerable [85]. This results in more efficient production, which increases rural households’ income. Additionally, the improved irrigation system generates opportunities in non-agricultural sectors as a multiplier effect [86]. The inconclusive relationship between irrigated land and RT2 found in the OLS model is supported by evidence suggesting that the effects of irrigation investment on non-farm employment in Bangladesh are indirect and complex. The findings above confirm Hypothesis 1, showing a positive effect of irrigation on both RT1 and RT2.

The share of irrigated land was positively linked to RT1 with a coefficient of 0.139 (p-value = 0.021) and RT2 with a coefficient of 0.358 (p-value = 0.003). Interaction terms were also positively linked to RT1 with a coefficient of 0.013 (p-value = 0.080) but not to RT2 with a coefficient of 0.003 (p-value = 0.772). There is evidence of a positive effect of irrigation on RT1 across stages of RT, with the direction of the effect remaining positive and statistically significant in all stages. A percentage increase in the proportion of irrigated land resulted in a 15.2% increase in RT1 in the early stages and up to 18.0% in the later stages. These findings imply that the effect of the increased share of irrigated land is more obvious in the later phases of RT, particularly for RT1. This finding is consistent with studies conducted in China and Pakistan [23,47]. In contrast, the impact of the share of irrigated land on RT2 across different stages of RT was inconclusive. This could be the result of inadequate irrigation infrastructure coverage and ineffective irrigation projects [87]. The findings support the second hypothesis.

The results indicate that in the FEM, the price ratio of high-valued to total agricultural commodities had a positive effect on RT1. However, the wage rate of rural workers in non-farm employment and the price index of agricultural inputs did not yield conclusive effects on RT1. A higher price ratio serves as a stimulus for farmers to prioritize the cultivation of high-value agricultural commodities such as vegetables, fruits, fish, and livestock. This incentive motivates them to allocate additional resources and exert greater effort toward cultivation, thereby augmenting their contribution to agricultural production [24]. It is plausible that farmers might be less likely to invest in irrigated land for high-value agriculture if the wage rate of rural labourers in non-farm employment is high, as they may earn more money from non-farm employment. Similarly, if the price index of agricultural inputs is high, farmers may be less inclined to invest in the cost of pumping irrigation water—typically through the rental market or pump hire—due to the high cost of production. For RT2, the wage rate of rural labor in non-farm employment showed a positive effect, while the price ratio of high-valued to total agricultural commodities and the price index of agricultural inputs did not provide any conclusive evidence. A positive effect on RT2 indicates that a higher wage rate in non-agricultural employment encourages rural workers to seek non-agricultural employment opportunities, resulting in a larger proportion of the labor force engaged in non-agricultural activities [24]. This is a sign of rural economic diversification, suggesting a preference for non-agricultural employment, which is perceived as more lucrative. This finding supports hypothesis 3.

The inclusion of district-fixed effects in the regression results (Table 3) strengthens the study`s findings. The coefficients of key variables, including ph_pwhole, Pinp_index, and NonFwage, show only minor changes with the incorporation of district effects, emphasizing the significance of district-specific factors in influencing rural transformation. Notably, NonFwage demonstrates a significant positive effect at the 1% level when district effects are considered, indicating considerable influences at the district level that were not captured in the model without these effects. Additionally, the coefficients for time-specific dummy variables (2000, 2005, 2010, 2016) are statistically significant, underscoring the importance of both temporal and district-specific factors in driving rural transformation.

We employed the Moment Quantile Regression (MM-QR) technique to examine the impact of irrigation at different points of the distribution of the RT, rather than just at the mean. This analysis provides a deeper understanding of how irrigation effects vary across different segments of the RT distribution, revealing the differential impact across quantiles in Table 4. The MM-QR approach is particularly suited for handling nonlinear relationships and is robust against outliers and heteroscedasticity, which increases the reliability of the findings, especially in the context of skewed distributions. Similarly, Saha et al. (2025) used the MM-QR approach for regional analysis [88]. The MM-QR analysis uncovers the nuanced impacts of irrigation on RT1 and RT2, offering insights crucial for policy design. For RT1, irrigation has a significant positive effect at the lower quantiles (25th and 50th), suggesting its strong influence in these segments. However, this influence becomes statistically insignificant at the higher quantiles (75th and 90th), indicating that other factors play a dominant role at these levels. In contrast, the effect of irrigation is positive and statistically significant on RT2 at the higher quantiles (50th, 75th, and 90th), while its impacts at the lowest quantile (25th) remain positive but insignificant These differing effects emphasize the importance of tailoring policy interventions to the specific needs of different groups, ensuring more targeted and effective outcomes.

**Table 4:** MM-QR with fixed effect results from the effect of irrigation on RT.

	(0.25)	(0.50)	(0.75)	(0.90)	(0.25)	(0.50)	(0.75)	(0.90)
Variables	RT1	RT1	RT1	RT1	RT2	RT2	RT2	RT2
IR_land	0.156^*^	0.142^**^	0.123	0.115	0.322	0.361^***^	0.385^***^	0.403^**^
	(0.055)	(0.018)	(0.176)	(0.314)	(0.194)	(0.005)	(0.005)	(0.032)
ph_pwhole	0.054	0.070^*^	0.092	0.101	0.091	0.117	0.133	0.145
	(0.334)	(0.095)	(0.142)	(0.200)	(0.563)	(0.152)	(0.125)	(0.225)
Pinp_index	0.006	0.024	0.052	0.063	−0.080	−0.032	−0.003	0.019
	(0.897)	(0.455)	(0.286)	(0.304)	(0.496)	(0.600)	(0.957)	(0.831)
NonFwage	−0.002	−0.007	−0.016	−0.019	0.104^***^	0.082^***^	0.068^***^	0.057^***^
	(0.848)	(0.297)	(0.135)	(0.151)	(0.000)	(0.000)	(0.000)	(0.004)
IR_D1	0.010	0.012^*^	0.016	0.017	0.004	0.003	0.003	0.003
	(0.252)	(0.061)	(0.117)	(0.178)	(0.879)	(0.778)	(0.796)	(0.855)
IR_D2	0.043^***^	0.041^***^	0.039^***^	0.039^***^	0.013	0.009	0.006	0.004
	(0.000)	(0.000)	(0.000)	(0.001)	(0.531)	(0.425)	(0.603)	(0.806)
*N*	128	128	128	128	128	128	128	128

*p*-values in parentheses: ^*^ *p* < 0.1, ^**^ *p* < 0.05, ^***^ *p* < 0.01

In Table 5, we present the results of our analysis of the impact of irrigation on per capita rural income and the share of irrigated land, with three control variables: the wage rate of rural labour in non-farm employment, the price ratio of high-valued to total agricultural commodities, and the price index of agricultural inputs. The results of OLS and FEM regressions suggest a positive effect of irrigation on rural income per capita. This finding is consistent with a study conducted in Fujian, Henan, and Sichuan provinces of China, which reported that irrigation positively impacted rural income [78]. Another study showed that irrigation had a positive effect on total per capita income in rural China, mainly due to its effect on crop income [20]. The R-squared values for the models were 0.901 (OLS) and 0.969 (FE), with an interaction term of 0.970 (FE) in the rural income equation. These values indicate that the model accurately represents the factors influencing rural income.

**Table 5:** The impact of irrigation on per capita rural income.

	OLS	FEM	
	(1)	(2)	(3)
	Ln rural per capita income	Ln rural per capita income	Ln rural per capita income
Irrigated Land	0.648^*^ (0.375)	2.405^***^(0.879)	1.631^*^ (0.950)
Pinp_index	0.660^*^(0.369)	−0.588 (0.423)	−0.428 (0.432)
NonFwage	0.613^***^ (0.119)	0.636^***^ (0.115)	0.628^***^ (0.114)
ph_ptotal	1.358^***^ (0.466)	1.578^**^ (0.644)	1.504^**^ (0.640)
IR*D1			0.210^**^ (0.103)
IR*D2			−0.017 (0.112)
Year effect of IR			
2000.		1.434 (0.103)	1.131(0.221)
2005.		1.661^**^(0.044)	1.337(0.128)
2010.		2.062^**^(0.010)	1.687^*^(0.052)
2016.		1.897^**^(0.018)	1.508^*^(0.086)
_cons	3.988^***^(0.307)	4.282^***^(0.374)	4.397^***^(0.393)
*N*	128	128	128
*R* ^2^	0.901	0.969	0.970

*Notes: Standard errors in parentheses;* ^*^ *p < 0.1,* ^**^ *p < 0.05,* ^***^ *p < 0.01*

In Table 5, considering the FEM with interaction terms to analyze rural income, irrigation only affected rural income in the early stages of RT. In the early stages of RT in Bangladesh, when agricultural output and infrastructure may be limited, irrigation may play a crucial role in increasing agricultural output and boosting agricultural productivity. This may lead to a higher rural income. Consequently, the impact of irrigation on rural income is substantial at the initial stage, which aligns with Shi’s study in China. However, in the later stages of RT in Bangladesh, access to markets, technology, and education may become more influential in determining rural income. Therefore, at a later stage, the impact of irrigation on rural income may be inconclusive because of these factors, which differs from the study in China and Pakistan [46,47]. These findings support hypothesis four.

From 2000 to 2016, the impact of irrigation on rural income in Bangladesh exhibited an evolving trend, as demonstrated by the changing coefficients and their statistical significance. In 2000, the coefficients 1.434 and 1.131 for FEM and FEM with an interaction term indicated that irrigation had a positive effect, although they were statistically insignificant. This suggests that the effects were not uniform across all areas. By 2005, the effect strengthened, with coefficients rising to 1.661 and 1.337, and the former became statistically significant at the 5% level. This improvement can be attributed to advancements in farming policies and irrigation technologies. The trend continued upward, reaching its peak in 2010, with coefficients of 2.062 and 1.687, signaling those years of investing in irrigation infrastructure had finally borne fruit. However, by 2016, although the coefficients remained positive and significant (1.897 and 1.508), there was a slight decline from the 2010 peak. This decline suggests a potential saturation of benefits from existing irrigation technologies and methods, highlighting the need for continuous innovation and policy adjustments to sustain, and enhance the economic impacts of irrigation on rural incomes. This comprehensive analysis underscores the importance of adapting irrigation practices over time to address evolving conditions and optimize their benefits.

### 4.7. Robustness check: Key variables and sequential inclusion of control variables

The robustness check for the effects of irrigation on rural transformation and rural income is presented in Tables 6–8. This check helps determine whether the relationship between the key variables and the dependent variable is stable or sensitive to changes in model specification. First, we test the key variables independently with the dependent variable as a baseline, showing how robust the key variables are without the confounding effects of control variables. Next, we add control variables one at a time to examine how each additional factor influences the relationship between the key variables and the outcome. This stepwise inclusion helps identify whether any control variable significantly modifies the results, suggesting that such factors could potentially confound the initial relationship. If the key variables maintain their significance and effect size after the inclusion of multiple control variables, it indicates that the results are robust.

**Table 6:** Robustness check of irrigation effects on RT1.

	(1)	(2)	(3)	(4)	(5)
	RT1	RT1	RT1	RT1	RT1
IR land	0.344^***^	0.265^***^	0.256^***^	0.320^***^	0.199^***^
	(0.000)	(0.000)	(0.000)	(0.000)	(0.000)
ph_ptotal		0.089^*^			
		(0.051)			
Pinp_index			0.058^*^		
			(0.080)		
NonFwage				0.005	
				(0.578)	
IR*D1					0.012^*^
					(0.079)
IR*D2					0.043^***^
					(0.000)
_cons	−0.004	−0.029^**^	−0.027^**^	−0.019	0.010^*^
	(0.280)	(0.030)	(0.049)	(0.488)	(0.083)
*N*	128	128	128	128	128
*R* ^2^	0.937	0.939	0.939	0.937	0.954

*Notes: p*-values in parentheses; ^*^ *p* < 0.1, ^**^ *p* < 0.05, ^***^ *p* < 0.01

**Table 7:** Robustness check of irrigation on RT2.

	(1)	(2)	(3)	(4)	(5)
	RT2	RT2	RT2	RT2	RT2
IR land	0.868^***^	0.582^***^	0.893^***^	0.382^***^	0.757^***^
	(0.000)	(0.000)	(0.000)	(0.000)	(0.000)
ph_ptotal		0.325^***^			
		(0.000)			
Pinp_index			−0.016		
			(0.802)		
NonFwage				0.094^***^	
				(0.000)	
IR*D1					0.015
					(0.342)
IR*D2					0.026
					(0.124)
_cons	0.137^***^	0.045^*^	0.144^***^	−0.168^***^	0.150^***^
	(0.000)	(0.072)	(0.000)	(0.000)	(0.000)
*N*	128	128	128	128	128
*R* ^2^	0.962	0.967	0.962	0.976	0.963

*p*-values in parentheses

* *p* < 0.1, ** *p* < 0.05, *** *p* < 0.01

**Table 8:** Robustness check of irrigation effects on rural per capita income.

	(1)	(2)	(3)	(4)	(5)
	Ln rural per capita income	Ln rural per capita income	Ln rural per capita income	Ln rural per capita income	Ln rural per capita income
IR land	6.184^***^	3.474^***^	6.866^***^	2.305^***^	4.887^***^
	(0.000)	(0.000)	(0.000)	(0.000)	(0.000)
ph_ptotal		3.073^***^			
		(0.000)			
Pinp_index			−0.448		
			(0.404)		
NonFwage				0.752^***^	
				(0.000)	
IR*D1					0.312^**^
					(0.016)
IR*D2					0.116
					(0.392)
_cons	6.563^***^	5.687^***^	6.741^***^	4.123^***^	6.775^***^
	(0.000)	(0.000)	(0.000)	(0.000)	(0.000)
*N*	128	128	128	128	128
*R* ^2^	0.949	0.958	0.949	0.966	0.952

*p*-values in parentheses

* *p* < 0.1, ** *p* < 0.05, *** *p* < 0.01

Table 6, like Table 3, indicates a positive correlation between the share of irrigated land and RT1. However, the coefficient in Table 6 is slightly higher at 0.344, which diminishes with the inclusion of control variables and ultimately stabilizes at approximately 0.199 in the comprehensive model. The relationship remains statistically significant in both tables. Table 3 shows a p-value for the FEM of less than 0.003, while in Table 6, the relationship continues to be significant at the 1% level across all model specifications, confirming the robustness of the irrigation effect on RT1. Although the coefficient values in Table 3 and Table 6 differ slightly, the main conclusion remains the same: irrigated land has a positive and significant effect on RT1, suggesting that investment in irrigation is essential to promote high-value agricultural goods. Babovic et al. (2009) also demonstrated the positive influence of irrigation on agricultural productivity in Serbia, significantly increasing the production of fruits and vegetables [89].

Both Table 3 and Table 7 show a positive relationship between the share of irrigated land and RT2, but the coefficient is substantially larger in Table 7 (0.868, without additional controls) compared to Table 3 (0.372). As control variables are added in Table 7, the coefficient decreases to 0.757, but it remains positive and significant in all specifications. This indicates that the positive impact of irrigation on RT2 is robust, although slightly diminished when other factors are considered. The relationship between irrigated land and RT2 is highly significant in both tables. In Table 3, the p-value is < 0.001, while in Table 7, the p-value is < 0.01 across all models, confirming the robustness of the relationship. This consistency across both tables suggests that irrigation positively influences RT2, and this effect holds even after the inclusion of control variables, which moderates the magnitude of the impact but does not undermine its statistical significance. Similarly, Angood et al. (2003) found that irrigation in Bangladesh plays a significant role in enhancing rural non-farm employment, further supporting this result [22].

As with RT1 and RT2, there is a positive and statistically significant link between irrigation and rural per capita income, as shown in both Table 5 (FEM) and Table 8. While the coefficients in Table 8 are larger than those in Table 5, they all indicate a positive effect of irrigation on rural income. In both tables, the relationship between irrigated land and rural income is significant. The FEM model in Table 5 shows a significant result (p-value < 0.01). In Table 8, the significance level remains at <0.01 for all models, demonstrating that the positive effect of irrigation on rural income is strong and stable across different model specifications. This result is consistent with the findings of Jatana & Tesfahun (2024, who showed that small-scale irrigation systems have a positive effect on rural income in Ethiopia [26].

The variations in coefficient magnitude across models in Tables 6–8 can be attributed to methodological differences in model specifications. Each model incorporates different variables, which influence the interpretation and magnitude of the “IR land” coefficients. For example, the inclusion of interaction terms (IRD1, IRD2) and additional control variables (ph_ptotal, Pinp_index, NonFwage) in the later models may redistribute the explained variance among the included variables, thereby altering the “IR land” coefficients. This is a common phenomenon in econometric analyses, where coefficients can vary depending on changes in model specification, especially when testing robustness across different configurations. The coefficients for the IR land variable decrease when the non-farm wage variable is included in the model (e.g., 0.868 in Model 1 and 0.382 in Model 4, Table 7; 6.184 in Model 1 and 2.305 in Model 4, Table 8). However, the sign of the coefficients remains consistently positive and statistically significant (p < 0.01) across all models. These findings reinforce the robustness of the baseline findings [90], indicating that irrigation continues to have a positive impact on RT2 and rural income, even when control variables such as non-farm wages are included in Model 4 (Tables 7 and 8). Although few models show variation in the magnitude of the coefficients, the direction and statistical significance of irrigation’s effect on RT and rural income remain consistent and robust.

## 5. Conclusions, limitations, and future research

This study highlights the significant potential of irrigation to enhance the livelihood of rural regions in Bangladesh and underscores the importance of allocating resources to irrigation to promote RT. Our findings indicate that irrigation substantially influences the production of high-value agricultural commodities, non-farm employment in rural areas, and per-capita rural income. However, the impact of irrigation varies across the stages of RT. We observed a positive effect of irrigation on high-value agricultural commodities in all stages of RT, suggesting that the benefits of irrigation are likely to be more pronounced in districts with an established agricultural infrastructure in Bangladesh. In contrast, the effect of irrigation on rural non-farm employment was inconclusive. For rural per capita income, a positive effect was observed only in the early phases of RT, with inconclusive results for later stages.

This highlights the potential role of other non-agricultural factors such as education, technology, infrastructure, access to markets, electricity, and access to credit in driving RT in Bangladesh. To optimize the advantages of irrigation for all indicators of RT, including high-value agricultural produce, non-agricultural employment, and rural income, we recommend integrating these non-agricultural factors alongside irrigation to strengthen RT in Bangladesh.

Moreover, to ensure the effective use of irrigation systems and maximize their benefits, it is crucial to invest not only in infrastructure but also in building water management skills at all levels. This includes training at the farm level, improving system management, and enhancing broader sectoral capacities. Investments in human capital will support sustainable irrigation practices and strengthen the resilience of rural communities to climate change and water scarcity.

Given the varying impact of irrigation across regions, it is crucial for the government to prioritize irrigation investment while considering regional priorities. District-level planning could help Bangladesh achieve its desired RT. Government policies such as the National Water Policy (1999) and the Integrated Water Resources Management Policy (2005) have historically facilitated irrigation and positively impacted rural livelihoods. Therefore, continued government support to enhance irrigated land, with a focus on regional requirements, is critical for regional rural transformation.

Several constraints limit the scope and depth of this analysis. Firstly, the data available from 2000 onwards may restrict a comprehensive understanding of the dynamic nature of rural transformation over a longer period. Secondly, the absence of irrigated land data across all sixty-four districts limits the research to the districts where this data is available. Additionally, since the HIES is updated only every five years, it makes it challenging to track ongoing trends and assess both short- and long-term changes in rural areas. While the HIES provides a wealth of information, it may not fully capture all factors driving rural change, as it lacks certain social and environmental variables. Moreover, as this study primarily focuses on quantitative data, it does not address qualitative aspects, such as sociocultural dynamics, which are essential to gain a holistic understanding of rural transformation.

Future research could explore the gendered and socioeconomic impacts of irrigation investment, particularly its effects on women and rural inequalities. Additionally, investigating the long-term sustainability of irrigation systems – including environmental impacts and climate change adaptation strategies – would be valuable. Incorporating qualitative data from farmers could provide deeper insights into the challenges faced in irrigation adoption. Further research on the role of the private sector could also inform policies aimed at encouraging sustainable investment in irrigation infrastructure.

## Supporting information

S1 Appendix T score and RT index of different district. (DOCX)
