# Supplementary material for: Effect of irrigation on rural transformation and income at the district level in Bangladesh
Source: PLoS One. 2025 Jun 18;20(6):e0326504. doi: 10.1371/journal.pone.0326504 (PMC12176131; doi:10.1371/journal.pone.0326504)
Supplement: S1 Appendix — (DOCX) [file pone.0326504.s001.docx]

**Appendix A**

**RT Score and RT Index of Different District**

| Year | District | RT score | RT index | Year | District | RT score | RT index |
| --- | --- | --- | --- | --- | --- | --- | --- |
| 2000 | Barguna | -1.78287 | Stage1 | 2000 | Magura | -1.31531 | Stage1 |
| 2005 | Barguna | -1.12331 | Stage1 | 2005 | Magura | -0.62787 | Stage2 |
| 2010 | Barguna | 0.068254 | Stage2 | 2010 | Magura | 0.338456 | Stage2 |
| 2016 | Barguna | 1.328037 | Stage3 | 2016 | Magura | 1.340367 | Stage3 |
| 2000 | Barisal | -1.23261 | Stage1 | 2000 | Maulvibazar | -1.58697 | Stage1 |
| 2005 | Barisal | -0.67842 | Stage2 | 2005 | Maulvibazar | -0.5858 | Stage2 |
| 2010 | Barisal | 0.360031 | Stage2 | 2010 | Maulvibazar | 1.026705 | Stage3 |
| 2016 | Barisal | 2.194541 | Stage3 | 2016 | Maulvibazar | 1.921949 | Stage3 |
| 2000 | Chandpur | -1.95599 | Stage1 | 2000 | Mymensingh | -0.61232 | Stage2 |
| 2005 | Chandpur | -1.1572 | Stage1 | 2005 | Mymensingh | 0.257808 | Stage2 |
| 2010 | Chandpur | -0.33192 | Stage2 | 2010 | Mymensingh | 1.472636 | Stage3 |
| 2016 | Chandpur | 0.809857 | Stage3 | 2016 | Mymensingh | 2.748399 | Stage3 |
| 2000 | Chittagong | -1.24509 | Stage1 | 2000 | Naogaon | -1.68454 | Stage1 |
| 2005 | Chittagong | -0.65591 | Stage2 | 2005 | Naogaon | -0.80908 | Stage1 |
| 2010 | Chittagong | 0.059051 | Stage2 | 2010 | Naogaon | 0.377827 | Stage2 |
| 2016 | Chittagong | 2.200873 | Stage3 | 2016 | Naogaon | 1.170586 | Stage3 |
| 2000 | Comilla | -1.64356 | Stage1 | 2000 | Natore | -1.47033 | Stage1 |
| 2005 | Comilla | -0.89058 | Stage1 | 2005 | Natore | -0.57806 | Stage2 |
| 2010 | Comilla | -0.03322 | Stage2 | 2010 | Natore | 0.900839 | Stage3 |
| 2016 | Comilla | 2.309533 | Stage3 | 2016 | Natore | 2.181837 | Stage3 |
| 2000 | Dhaka | -0.25267 | Stage2 | 2000 | Noakhali | -1.53657 | Stage1 |
| 2005 | Dhaka | 0.690936 | Stage2 | 2005 | Noakhali | -1.00541 | Stage1 |
| 2010 | Dhaka | 1.933319 | Stage3 | 2010 | Noakhali | -0.21993 | Stage2 |
| 2016 | Dhaka | 3.056172 | Stage3 | 2016 | Noakhali | 1.065778 | Stage3 |
| 2000 | Dinajpur | -1.7624 | Stage1 | 2000 | Pabna | -0.93449 | Stage1 |
| 2005 | Dinajpur | -1.10658 | Stage1 | 2005 | Pabna | -0.04186 | Stage2 |
| 2010 | Dinajpur | -0.06112 | Stage2 | 2010 | Pabna | 1.255547 | Stage3 |
| 2016 | Dinajpur | 1.539708 | Stage3 | 2016 | Pabna | 1.97634 | Stage3 |
| 2000 | Faridpur | -0.9154 | Stage1 | 2000 | Patuakhali | -1.67179 | Stage1 |
| 2005 | Faridpur | -0.10327 | Stage2 | 2005 | Patuakhali | -1.00569 | Stage1 |
| 2010 | Faridpur | 1.188471 | Stage3 | 2010 | Patuakhali | -0.17907 | Stage2 |
| 2016 | Faridpur | 2.255477 | Stage3 | 2016 | Patuakhali | 1.657903 | Stage3 |
| 2000 | Gaibandha | -1.40346 | Stage1 | 2000 | Rajshahi | -0.71294 | Stage2 |
| 2005 | Gaibandha | -0.76002 | Stage2 | 2005 | Rajshahi | 0.159793 | Stage2 |
| 2010 | Gaibandha | 0.09547 | Stage2 | 2010 | Rajshahi | 1.695094 | Stage3 |
| 2016 | Gaibandha | 1.520455 | Stage3 | 2016 | Rajshahi | 2.112387 | Stage3 |
| 2000 | Jessore | -1.01925 | Stage1 | 2000 | Rangpur | -1.19818 | Stage1 |
| 2005 | Jessore | -0.25841 | Stage2 | 2005 | Rangpur | -0.50934 | Stage2 |
| 2010 | Jessore | 0.719617 | Stage3 | 2010 | Rangpur | 0.32578 | Stage2 |
| 2016 | Jessore | 2.101726 | Stage3 | 2016 | Rangpur | 1.79511 | Stage3 |
| 2000 | Khagrachhari | -2.14678 | Stage1 | 2000 | Satkhira | -1.88265 | Stage1 |
| 2005 | Khagrachhari | -1.46379 | Stage1 | 2005 | Satkhira | -0.83966 | Stage1 |
| 2010 | Khagrachhari | -0.69254 | Stage2 | 2010 | Satkhira | 0.744054 | Stage3 |
| 2016 | Khagrachhari | 0.603807 | Stage2 | 2016 | Satkhira | 2.270591 | Stage3 |
| 2000 | Khulna | -0.42723 | Stage2 | 2000 | Shariatpur | -2.12979 | Stage1 |
| 2005 | Khulna | -0.0114 | Stage2 | 2005 | Shariatpur | -1.5566 | Stage1 |
| 2010 | Khulna | 1.002205 | Stage3 | 2010 | Shariatpur | -0.57507 | Stage2 |
| 2016 | Khulna | 2.884727 | Stage3 | 2016 | Shariatpur | 0.819546 | Stage3 |
| 2000 | Kishoreganj | -2.06865 | Stage1 | 2000 | Sirajganj | -1.89349 | Stage1 |
| 2005 | Kishoreganj | -1.07056 | Stage1 | 2005 | Sirajganj | -0.78353 | Stage1 |
| 2010 | Kishoreganj | -0.11977 | Stage2 | 2010 | Sirajganj | 0.697542 | Stage3 |
| 2016 | Kishoreganj | 0.912369 | Stage3 | 2016 | Sirajganj | 1.901577 | Stage3 |
| 2000 | Kurigram | -1.28413 | Stage1 | 2000 | Sunamganj | -2.17827 | Stage1 |
| 2005 | Kurigram | -0.56349 | Stage2 | 2005 | Sunamganj | -1.38591 | Stage1 |
| 2010 | Kurigram | 0.360388 | Stage2 | 2010 | Sunamganj | -0.09994 | Stage2 |
| 2016 | Kurigram | 1.297783 | Stage3 | 2016 | Sunamganj | 0.72203 | Stage3 |
| 2000 | Kushtia | -1.90063 | Stage1 | 2000 | Sylhet | -0.41153 | Stage2 |
| 2005 | Kushtia | -1.28763 | Stage1 | 2005 | Sylhet | 0.48877 | Stage2 |
| 2010 | Kushtia | -0.4937 | Stage2 | 2010 | Sylhet | 1.536766 | Stage3 |
| 2016 | Kushtia | 0.44418 | Stage2 | 2016 | Sylhet | 2.755221 | Stage3 |
| 2000 | Lalmonirhat | -1.84603 | Stage1 | 2000 | Tangail | -1.40878 | Stage1 |
| 2005 | Lalmonirhat | -1.20755 | Stage1 | 2005 | Tangail | -0.93265 | Stage1 |
| 2010 | Lalmonirhat | -0.42808 | Stage2 | 2010 | Tangail | -0.118 | Stage2 |
| 2016 | Lalmonirhat | 0.859998 | Stage3 | 2016 | Tangail | 1.352403 | Stage3 |
